# Determinants of arterial stiffness in patients with type 2 diabetes mellitus: a cross sectional analysis

**DOI:** 10.1038/s41598-023-35589-4

**Published:** 2023-06-02

**Authors:** Mawadah Staef, Christian Ott, Dennis Kannenkeril, Kristina Striepe, Mario Schiffer, Roland E. Schmieder, Agnes Bosch

**Affiliations:** 1 Department of Nephrology and Hypertension, Friedrich-Alexander-University Erlangen-Nürnberg (FAU), Ulmenweg 18, 91054 Erlangen, Germany; 2 Paracelsus Medical School Nuremberg, Nuremberg, Germany

**Keywords:** Diseases, Medical research, Risk factors

## Abstract

In patients with type 2 diabetes mellitus (T2DM) arterial stiffness is associated with increased cardiovascular and total mortality. Little is known about determinants of arterial stiffness in clinical routine. Identification of potential determinants of arterial stiffness will help to address treatment targets for patients in the early state of T2DM. This is a cross-sectional analysis of arterial stiffness in 266 patients in the early stage of T2DM who did not have cardiovascular or renal complications. Parameters of arterial stiffness such as central systolic blood pressure (cSBP), central pulse pressure (cPP) and pulse wave velocity (PWV) were measured with the SphygmoCor System (AtCor Medical). We investigated the influence of parameters of glucose metabolism, lipid status, body constitution, blood pressure (BP) and inflammation on the stiffness parameters using multivariate regression analysis. The study cohort consisted of male and female patients aged 61 ± 8 years with mean diabetes duration of 6.4 ± 5.1 years, mean HbA1c 7.1 ± 0.9%, mean cSBP 121 ± 12 mmHg, mean cPP 44 ± 10 mmHg and mean PWV 8.9 ± 1.8 m/s. Multiple regression analysis identified waist circumference (WC) (beta = 0.411, p = 0.026), LDL-cholesterol (beta = 0.106, p = 0.006), systolic office BP (beta = 0.936, p < 0.001) and diabetes duration (beta = 0.233, p = 0.043) as potential determinants of cSBP. cPP was determined by sex (beta = 0.330, p = 0.008), age (beta = 0.383, p < 0.001), systolic office BP (beta = 0.370, p < 0.001) and diabetes duration (beta = 0.231, p = 0.028) whereas for PWV the following determinants could be identified: age (beta = 0.405, p < 0.001), systolic office BP (beta = 0.421, p < 0.001) and diabetes duration (beta = 0.073, p = 0.038). In addition to the known parameters age, sex and systolic office BP serum LDL-cholesterol, WC and diabetes duration have been identified as determinants of arterial stiffness in patients with T2DM. Treatment of patients in the early stage of T2DM should focus on these clinical parameters to prevent progression of arterial stiffness and as a consequence reduce cardiovascular mortality.

Trial registration: The patients included in the analysis participated in one of the following clinical trials NCT02752113 (registered 26.4.2016), NCT02383238 (09.03.2015), NCT02471963 (15.06.2015), NCT01319357 (21.03.2011) (http://www.clinicaltrials.gov).

## Introduction

Vascular changes represent a key prognostic factor in patients with type 2 diabetes mellitus (T2DM). The idea of palpating the wrist, a time honored tradition in medicine, goes back to antiquity and was used for centuries to estimate the health of the circulation^1^. In the past decades several devices have been developed and are clinically used to estimate arterial stiffness in humans^1^. It has been recognized in the 2018 ESC/ESH guidelines on the management of arterial hypertension, that arterial stiffness, measured using pulse wave velocity (PWV) and pulse wave analysis (PWA) is an established intermediate surrogate endpoint of vascular dysfunction^2^. The effect of diabetes on arterial stiffness has been shown to equal in its magnitude 6–15 years of chronological aging on vessels^3,4^. In patients with T2DM arterial stiffness has been shown to predict cardiovascular events independent of traditional risk factors such as glycemic control and 24 h ambulatory blood pressure (BP)^5^. Moreover, arterial stiffness has been repeatedly shown to predict cardiovascular and all-cause mortality in patients with high cardiovascular risk profile^6^ and especially in patients with T2DM^7–9^. The REBOUND study, a large multicenter prospective observational cohort study with a median follow-up of 8.6 years, demonstrated that increased arterial stiffness measured by PWV predicts the risk of cardiovascular and all-cause mortality in patients with T2DM^9^.

However, the precise pathological mechanism how T2DM increases vascular stiffness in still unknown. Previous data suggest that increased arterial stiffness provides a potential mechanistic link between vascular changes and the development of diastolic dysfunction, a typical target organ damage in T2DM^1^. Arterial stiffness is known to cause premature wave reflection leading to increased mid-to-late systolic load and thus left ventricular diastolic dysfunction^10^. The following parameters determining arterial stiffness have been previously investigated: (1) Age: Several animal and human studies demonstrated that large elastic arteries, such as the aorta, show increases in arterial stiffness with aging, which correlates with histological and biochemical changes within the arterial wall^4^. (2) High blood pressure (BP): High BP is known to cause vascular damage and elastin fragmentation, which leads to increased arterial stiffness^4^. (3) Sex: Interestingly, vascular stiffening is sexually dimorphic. Whereas the age-related increases in vascular stiffness are less in women before menopause than those in age-matched men, after menopause woman show a greater increase in vascular stiffness compared to men^11^. Besides age, sex and BP other mechanisms have been proposed to mediate vascular stiffness especially in patients with T2DM: Non-enzymatic advanced glycation of proteins (AGEs), increased oxidative stress, increased incidence of atherosclerosis and displayed endothelial dysfunction. In summary, the current literature identified age, sex and systolic BP as determinants of arterial stiffness. However, there is a lack of analysis of further treatment target parameters related to vascular stiffness in patients with T2DM such as parameters of body shape [BMI, waist circumference (WC)] glucose control (HbA1c), blood lipids (serum LDL-cholesterol, serum triglycerides, serum Non-HDL-cholesterol), parameters of inflammation (hsCRP) and diabetes duration. Identification of determinants of arterial stiffness might help to address further treatment targets in patients in the early stage of T2DM. In addition, patients with yet unknown very high cardiovascular risk might be identified. Thus, treatment might be initiated earlier and the progression of arterial stiffness and as a consequence cardiovascular mortality reduced. Our cross-sectional analysis explores these potential determinants of arterial stiffness in a cohort of 266 patients in the early stage of T2DM.

## Methods

### Study design

This is a cross-sectional analysis of baseline parameters of patients in the early state of T2DM, who participated in one of the following randomized clinical trials (http://www.clinicaltrials.gov) during 2005–2021: “Effects of Empagliflozin + Linagliptin vs Metformin + Insulin Glargine on Renal and Vascular Changes in Type 2 Diabetes (ELMI)” (NCT02752113), “Effect of Dapagliflozin on Microvascular and Macrovascular Circulation and Total Body Sodium Content (Dapa)” (NCT02383238), “Effect of Empagliflozin on Macrovascular and Microvascular Circulation and on Endothelium Function (EMPA)” (NCT02471963), “Effects of Saxagliptin on Endothelial Function (ESENDI)” (NCT01319357). The clinical trials were conducted at the Clinical Research Centre of the Department of Nephrology and Hypertension, University Hospital Erlangen-Nuremberg, Germany (www.crc-erlangen.de). The patients were recruited from the University outpatient clinic, local newspaper advertisement and referring physicians. The study protocols have been approved by the local Ethics Committee of the University of Erlangen-Nuremberg. Before study inclusion written informed consent was obtained from each subject. The studies were conducted according to the principles of good clinical practice guidelines and tenets of the Declaration of Helsinki.

### Study population

The study population consisted of patients aged 28–76 years with T2DM and without overt end-organ damage. All patients fulfilled the following criteria: No treatment with insulin, HbA1c < 11% or fasting plasma glucose < 240 mg/dl, eGFR > 50 ml/min/1.73 m^2^, no cardiovascular event within the last 3 months. 166 out of 266 patients had diagnosed hypertension and 112 out of 266 patients hypercholesterinemia.

### Assessment of vascular stiffness

Blood pressure and heart rate were measured according to European Society of Hypertension/European Society of Cardiology guideline recommendations in standard fashion by validated devices in a seated position after 5 min of rest^2^.

The non‐invasive assessment of central aortic pulse wave and PWV was performed in a quiet temperature-controlled examination room with the patient being in supine position. The SphygmoCor XCEL System (AtCor Medical, Sydney, Australia) was used to assess parameters of vascular stiffness under resting conditions. Details about the system have been previously published^12–14^. In brief, the system is a validated highly reliable method with coefficient of variation below 10%, which allows the calculation of central BP based on brachial artery waveforms^15,16^. Peripheral brachial BP is measured with a conventional brachial oscillometric device. The device records the volumetric displacement related to the volume of the brachial artery within the cuff around the upper arm. Afterwards the system calculates the central aortic pressure wave from the peripheral signal by a validated transfer function^17^. This allows the measurement of the parameters cSBP and cDBP, cPP, augmentation pressure, augmentation index (normalized to a heart rate of 75 beats per minute), forward and reflected pressure pulse height.

Carotid-femoral pulse wave velocity (PWV) is the gold-standard to measure arterial stiffness non-invasively. The SpygmoCor XCEL system allows the validated determination of the aortic PWV. The system simultaneously uses the carotid pulse acquired by applanation tonometry and the femoral pulse acquired by a femoral cuff around the upper thigh. The software uses the foot‐to‐foot transit time between carotid and femoral pulse divided by the physical distance measured to calculate the PWV. Details about the method have been previously published^18,19^.

Vascular parameters under ambulatory conditions (24-h ABPM) such as 24-h cSBP, 24-h augmentation index and 24-h PWV were assessed using the Mobilograph™ (IEM, Stollberg, Germany)^20^.

### Statistical analysis

Normal distribution of data was confirmed by histogram and Kolmogorov–Smirnov test prior to further analysis. Data were expressed as mean ± standard deviation (SD) in text and tables. A two-sided p-value < 0.05 was considered statistically significant.

Determinants of arterial stiffness have been evaluated in three steps. First, multivariate regression analysis was performed including the parameters sex, age, BMI, WC, systolic office BP, HbA1c, serum LDL-cholesterol, serum triglycerides, hsCRP and diabetes duration. Vascular stiffness parameters entered our model as an independent variable. cSBP (office and 24-h ABPM), cPP (office and 24-h ABPM), and PWV (office and 24-h ABPM). A separate multiple regression analysis was performed for each of the six independent variables mentioned. Potential collinearity between the dependent variables in our model has been excluded by calculating correlation coefficients between the dependent variables.

Secondly, the study population has been separated into different groups based on the median of the following parameters: age, BMI, WC, systolic office BP, HbA1c, serum LDL-cholesterol, serum triglycerides, hsCRP and diabetes duration. Then unpaired t-test has been performed for the parameters cSBP (office and 24-h ABPM), cPP (office and 24-h ABPM), and PWV (office and 24-h ABPM), comparing parameters ≥ median to < median of the mentioned parameters.

Thirdly, bivariate correlation analyses were performed using Pearson’s test and partial correlation was used to adjust for confounding parameter age and systolic office BP.

All analyses were performed using IBM SPSS Statistics 22 (SPSS Inc, Chicago, IL/USA).

### Ethics approval and consent to participate

The study protocols have been approved by the local Ethics Committee of the University of Erlangen-Nuremberg. Before study inclusion written informed consent was obtained from each subject. The studies were conducted according to the principles of good clinical practice guidelines and tenets of the Declaration of Helsinki.

## Results

### General characteristics of the study population

The study cohort consisted of male and female patients with mean age of 61 ± 8 years, mean diabetes duration of 6.4 ± 5.1 years and mean HbA1c 7.1 ± 0.9%. 220 out of 266 patients (86.5%) took antidiabetic medication, such as metformin (220 patients, 84.6%), DPP4-I (14 patients, 5.3%) or sulfonylurea (4 patients, 1.5%). Further clinical baseline parameters of the study population are shown in Table 1.

**Table 1:** Baseline characteristics of the study population.

General parameters	
Age (years)	61.0 ± 8.1
Diabetes duration (years)	6.4 ± 5.1
Waist circumference (cm)	105 ± 11
BMI (kg/m^2^)	30.5 ± 4.2
Sex (m/f)	174/92
Mean blood pressure parameters under office condition	
Heart rate (bpm)	70 ± 9.6
Peripheral systolic BP (mmHg)	132 ± 13
Peripheral diastolic BP (mmHg)	77 ± 7.9
Peripheral pulse pressure (mmHg)	56 ± 11
Central systolic BP (mmHg)	121 ± 12
Central diastolic BP (mmHg)	78 ± 7.9
Central pulse pressure (mmHg)	44 ± 11
Central Augmentation pressure (mmHg)	12 ± 5.9
Central Augmentation index (–)	28 ± 9.1
Central Augmentation index @ heart rate of 75 bpm (–)	24 ± 8.8
Forward pulse pressure height (mmHg)	31 ± 6.3
Reflected pulse pressure height (mmHg)	20 ± 5.1
Pulse wave velocity (m/sec)	8.9 ± 1.8
Mean blood pressure parameters under 24-h ambulatory conditions	
Heart rate (bpm)	75 ± 9.8
Peripheral systolic BP (mmHg)	130 ± 11
Peripheral diastolic BP (mmHg)	79 ± 7.7
Pulse wave velocity (m/s)	8.8 ± 1.2
Central systolic BP (mmHg)	120 ± 8.9
Central diastolic BP (mmHg)	82 ± 7.1
Central pulse pressure (mmHg)	50 ± 8.7
Parameters of diabetes control	
Fasting plasma glucose (mg/dl)	144 ± 32
HbA1c (%)	7.1 ± 0.9
Lipids	
Cholesterol (mg/dl)	200 ± 38
HDL-cholesterol (mg/dl)	47 ± 11
LDL-cholesterol (mg/dl)	135 ± 30
Triglycerides (mg/dl)	169 ± 89
Non-HDL-cholesterol (mg/dl)	153 ± 35
Inflammation	
hsCRP (mg/l)	2.4 ± 2.7
Kidney	
Serum creatinine (mg/dl)	0.82 (0.71–0.92)
eGFR (CKD-EPI)	91 ± 11
UACR (mg/g creatinine)	22.1 ± 35

Data are presented as mean ± SD for normal distribution and as median (interquartile range) for not normal distributed data. *hsCRP* high sensitive C-reactive-protein, *eGFR* estimated glomerular filtration rate according to CKD-EPI formula, *UACR* urine albumin creatinine ratio.

Mean WC and body mass index (BMI) were elevated in the study population (Table 1). 166 out of 266 patients had previously diagnosed hypertension. Antihypertensive medication was frequent: 67 patients took one antihypertensive substance, 49 patients two substances, 36 patients three substances and 14 patients four or more antihypertensive substances. The following classes of antihypertensive substances were represented: ACE-I/ARB: 136 patients (51.1%), diuretics: 65 patients (24.4%), betablocker: 57 patients (21.4%), calcium antagonists: 65 patients (24.4%), aldosterone antagonists: 6 patients (2.3%). Mean BP under office conditions was 132 ± 13/77 ± 7.9 mmHg and under 24-h ambulatory conditions 130 ± 11/79 ± 7.7 mmHg. Parameters of central BP under office and 24-h conditions are shown in Table 1.

112 out of 266 patients had diagnosed hypercholesterinemia, whereas 79 out of the 112 patients with hypercholesterinemia took statins. Mean LDL-cholesterol and mean triglycerides of the study population indicate that dyslipidemia was still present in many patients (Table 1).

### Multiple regression analysis

**Figure 1:**
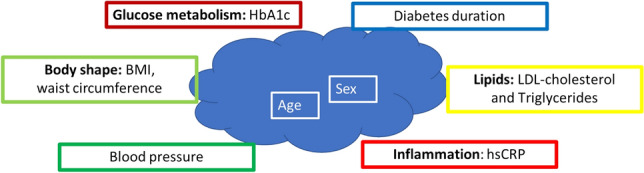
Systematic overview of clinical determinants assessed in relation to arterial stiffness.

Multiple regression analysis was performed for independent parameters of pulse wave analysis [cSBP (Table 2) and cPP (Table 3)] and for PWV (Table 4) both under office and 24-h ambulatory conditions. The description focuses on modifiable risk factors such as factors determining body shape (BMI, waist circumference), diabetes control (HbA1c, diabetes duration), lipid metabolism (LDL-cholesterol, triglycerides), blood pressure (systolic OBP) and inflammation (hsCRP). Figure 1 shows an overview of the parameters integrated in the multiple regression analysis as dependent parameters. For the full panel of potential determinants calculated please see Tables 2, 3, 4, which also include age and sex as non-modifiable factors.

**Table 2:** Results of multivariate regression analysis with dependent variable central systolic blood pressure.

Variable	Office conditions		24-h ambulatory conditions	
	Beta	p-value	Beta	p-value
Sex	− 0.082	0.075	0.138	0.299
Age	0.001	0.997	− 0.074	0.495
BMI	0.090	0.115	− 0.145	0.384
Waist circumference	− 0.081	0.190	**0.411**	**0.026**
HbA1c	− 0.028	0.406	− 0.015	0.878
LDL-cholesterol	**0.106**	**0.006**	**0.233**	**0.036**
Triglycerides	− 0.027	0.477	0.025	0.820
hsCRP	0.019	0.605	− 0.060	0.562
Systolic OBP	**0.936**	**< 0.001**	**0.429**	**< 0.001**
Diabetes duration	0.042	0.286	**0.233**	**0.043**

Significant values are in bold.

*BMI* body mass index, *LDL* low density lipid, *hsCRP* high sensitive C-reactive protein, *OBP* office blood pressure.

**Table 3:** Results of multivariate regression analysis with dependent variable central pulse pressure.

Variable	Office conditions		24-h ambulatory conditions	
	Beta	p-value	Beta	p-value
Sex	0.054	0.542	**0.330**	**0.008**
Age	**0.383**	**< 0.001**	**0.262**	**0.010**
BMI	0.058	0.597	− 0.045	0.768
Waist circumference	− 0.160	0.183	0.274	0.097
HbA1c	− 0.065	0.324	0.003	0.976
LDL-cholesterol	− 0.100	0.171	0.015	0.884
Triglycerides	0.068	0.364	0.062	0.549
hsCRP	− 0.001	0.992	− 0.086	0.372
Systolic OBP	**0.682**	**< 0.001**	**0.370**	**< 0.001**
Diabetes duration	0.021	0.780	**0.231**	**0.028**

Significant values are in bold.

*BMI* body mass index, *LDL* low density lipid, *hsCRP* high sensitive C-reactive protein, *OBP* office blood pressure.

**Table 4:** Results of multivariate regression analysis with dependent variable pulse wave velocity.

Variable	Office conditions		24-h ambulatory conditions	
	Beta	p-value	Beta	p-value
Sex	− 0.192	0.118	0.041	0.315
Age	**0.405**	**< 0.001**	**0.914**	**< 0.001**
BMI	0.019	0.903	− 0.052	0.306
Waist circumference	− 0.032	0.845	0.107	0.055
HbA1c	− 0.030	0.735	− 0.020	0.502
LDL-cholesterol	0.048	0.633	0.044	0.189
Triglycerides	0.117	0.263	0.003	0.917
hsCRP	0.051	0.596	− 0.023	0.467
Systolic OBP	**0.421**	**< 0.001**	**0.146**	**< 0.001**
Diabetes duration	0.018	0.862	**0.073**	**0.038**

Significant values are in bold.

*BMI* body mass index, *LDL* low density lipid, *hsCRP* high sensitive C-reactive protein, *OBP* office blood pressure.

Beyond age and sex the following parameters emerged as determinants:

- Pulse wave analysis parameters cSBP and cPP under 24-h ambulatory conditions: diabetes duration, systolic office BP, WC (only for cSBP), serum LDL-cholesterol (only for cSBP) (Tables 2, 3)
- Pulse wave analysis parameters cSBP and cPP under office conditions: systolic office BP and LDL-cholesterol (only for cSBP) (Tables 2, 3).
- Pulse wave velocity under 24 h ambulatory conditions: systolic office BP, diabetes duration (Table 4)
- Pulse wave velocity under office conditions: systolic office BP (Table 4)

### Stiffness parameters based on the median of potential determinants

In addition, the study population was divided according to the median of the potential determinants entered in the multiple regression analysis (Table 5). For pulse wave analysis under 24-h ambulatory conditions the following parameters beyond age revealed significant differences between the groups: serum triglycerides and systolic office BP (Table 5A,B). For pulse wave analysis under office conditions only systolic office BP emerged as significant determinant (Table 5A,B). The same was true for pulse wave velocity under 24 h ambulatory conditions as well as office conditions (Table 5C).

**Table 5:** Comparison of arterial stiffness parameter between different subgroups separated according to median of different variables.

	Variable	age	BMI	WC	HbA1c	LDL-c	TAG	hsCRP	sOBP	DDM
A: 24-h and office central systolic blood pressure (mmHg)										
24-h cSBP	≥ Median	119 ± 9.5	120 ± 8.7	120 ± 8.4	120 ± 8.9	121 ± 8.5	121 ± 8.6	120 ± 7.6	123 ± 9.3	120 ± 8.7
	< Median	120 ± 8.4	119 ± 9.3	119 ± 9.4	119 ± 8.9	119 ± 9.4	118 ± 9.1	120 ± 10.3	116 ± 6.9	119 ± 9.3
	p-value	0.470	0.660	0.373	0.264	0.245	**0.035**	0.993	**< 0.001**	0.829
Office cSBP	≥ Median	123 ± 13	121 ± 12	122 ± 12	122 ± 12	122 ± 12	122 ± 12	123 ± 9.1	129 ± 9,2	122 ± 12
	< Median	119 ± 10	120 ± 12	120 ± 12	120 ± 12	120 ± 12	120 ± 12	121 ± 10.2	112 ± 7.1	120 ± 11
	p-value	**0.016**	0.608	0.295	0.141	0.208	0.106	0.188	**< 0.001**	0.147
B: 24-h central pulse pressure (mmHg)										
24-h cSBP	≥ Median	51.4 ± 8.9	50.6 ± 8.1	50.1 ± 7.8	50.2 ± 8.7	50.7 ± 9.2	52.0 ± 8.7	49.5 ± 8.4	53.3 ± 9.1	51.4 ± 9.2
	< Median	48.4 ± 8.3	49.4 ± 9.3	50.3 ± 9.5	49.7 ± 8.9	49.5 ± 8.3	48.3 ± 8.4	50.1 ± 8.9	46.7 ± 6.9	48.2 ± 7.8
	p-value	**0.024**	0.398	0.962	0.722	0.362	**0.006**	0.751	**< 0.001**	**0.015**
Office cSBP	≥ Median	46.4 ± 10.3	44.1 ± 10.c6	44 ± 10	44.5 ± 9.9	43.9 ± 10	44.5 ± 10	44.1 ± 7.9	49.0 ± 9.8	45.2 ± 10
	< Median	41.1 ± 10.3	43.4 ± 10.4	44 ± 11	42.8 ± 11.1	43.5 ± 11	43.0 ± 11	43.8 ± 8.6	38.5 ± 8.1	42.2 ± 11
	p-value	**< 0.001**	0.618	0.997	0.215	0.714	0.264	0.854	**< 0.001**	**0.018**
C: 24-h pulse wave velocity										
24-h cSBP	≥ Median	9.7 ± 0.8	8.6 ± 1.1	8.9 ± 1.2	8.9 ± 1.3	8.8 ± 1.2	8.8 ± 1.1	8.8 ± 1.2	9.1 ± 1.3	9.1 ± 1.3
	< Median	7.8 ± 0.8	9.0 ± 1.3	8.8 ± 1.3	8.7 ± 1.2	8.9 ± 1.3	8.8 ± 1.3	9.0 ± 1.3	8.6 ± 1.1	8.5 ± 1.2
	p-value	**< 0.001**	0.042	0.529	0.377	0.649	0.940	0.406	**0.006**	**0.007**
Office cSBP	≥ Median	9.4 ± 1.9	8.8 ± 1.7	9.0 ± 1.9	8.7 ± 1.6	8.9 ± 1.9	9.1 ± 1.8	8.4 ± 1.3	9.5 ± 1.9	8.9 ± 1.7
	< Median	8.3 ± 1.4	8.9 ± 1.9	8.7 ± 1.7	9.0 ± 2.0	8.7 ± 1.9	8.7 ± 1.8	8.3 ± 1.6	8.2 ± 1.4	8.9 ± 1.8
	p-value	**< 0.001**	0.518	0.186	0.177	0.321	0.069	0.757	**< 0.001**	0.818

Significant values are in bold.

Data are presented as mean ± SD, *BMI* body mass index, *WC* waist circumference, *LDL-c* serum LDL-cholesterol, *TAG* serum triglycerides, *hsCRP* high sensitive C-reactive protein, *cSBP* central systolic blood pressure, *DDM* duration of diabetes mellitus.

### Correlations

#### Age

Patients’ age correlated with cSBP (r = 0.126, p = 0.041), cPP (r = 0.306, p < 0.001) and PWV (r = 0.395, p < 0.001).

#### BMI

There was a correlation between BMI and cPP (r = 0.127, p = 0.039), which persisted after adjustment for age (r = 0.185, p = 0.003) as well as after adjustment for age and systolic office BP (r = 0.133, p = 0.034) and cSBP (r = 0.078, p = 0.204, after adjustment for age: r = 0.161, p = 0.010), but not PWV (r = 0.027, p = 0.663).

#### WC

There was a correlation between WC and PWV (r = 0.110, p = 0.080, after adjustment for age: r = 0.145, p = 0.022), but not with cSBP (r = 0.045, p = 0.473) and cPP (r = − 0.028, p = 0.657).

#### HbA1c

There was no correlation between HbA1c and cSBP (r = 0.045, p = 0.464), cPP (r = 0.055, p = 0.371) and PWV (r = 0.037, p = 0.646).

#### Diabetes duration

There was a correlation between diabetes duration and PWV (r = 0.326, p < 0.001, which did not persist after adjustment for age (r = 0.085, p = 0.289). No correlation was present between diabetes duration and cSBP (r = 0.048, p = 0.434) as well as cPP (r = 0.101, p = 0.102).

#### Lipids

There was a correlation between serum LDL-cholesterol and cSBP (r = 0.125, p = 0.045), which persisted after adjustment for age (r = 0.136, p = 0.027) as well as PWV (r = 0.145, p = 0.020), which also persisted after adjustment for age (r = 0.190, p = 0.002) and age and systolic office BP (r = 0.142, p = 0.024)). There was no correlation between LDL-cholesterol and cPP (r = 0.089, p = 0.147). Serum triglyceride concentration correlated with PWV (r = 0.158, p = 0.011, age adjusted r = 0.210, p = 0.001, age and systolic office BP adjusted: r = 0.182, p = 0.004), but there was no correlation with cSBP and cPP. Serum non-HDL-cholesterol correlated with PWV (r = 0.137, p = 0.028, age adjusted r = 0.188, p = 0.003, age and systolic office BP adjusted 0.135, p = 0.032) and cSBP (r = 0.134, p = 0.030, age adjusted r = 0.188, p = 0.026). No correlation was present between non-HDL-cholesterol and cPP (r = 0.071, p = 0.251).

#### hsCRP

No correlation was present between hsCRP and cSBP (r = 0.157, p = 0.128) as well as cPP (r = 0.100, p = 0.337) and PWV (r = 0.112, p = 0.283).

#### Systolic office BP

There was a correlation between systolic office BP and cSBP (r = 0.684, p < 0.001, age adjusted: r = 0.698, p < 0.001) as well as cPP (r = 0.502, p < 0.001, age adjusted: r = 0.504, p < 0.001) and PWV (r = 0.404, p < 0.001, age adjusted: r = 0.382, p < 0.001).

### Sub-group-analysis in patients with hypercholesterinemia

A sub-group-analysis was performed comparing parameters of arterial stiffness in patients with diagnosed hypercholesterinemia (n = 112) compared to patients without diagnosed hypercholesterinemia (n = 154). There was a significant difference in baseline parameters between both groups: age, LDL-cholesterol, hsCRP (Table 6). No difference was found for the baseline parameters of office BP, heart rate, diabetes duration and eGFR between the groups (Table 6). Patients with T2DM and hypercholesterinemia showed higher PWV (9.1 ± 1.1 m/s) compared to patients without hypercholesterinemia (8.6 ± 1.3 m/s, p = 0.007). However, when adjusting for the baseline parameters the difference in PWV did not remain significant (p = 0.644). There was no difference in cSBP (119 ± 9.7 vs. 120 ± 8.3 mmHg, p = 0.260, p_adj_ = 0.272) and cPP (50 ± 8.9 vs. 50 ± 8.6 mmHg, p = 0.759, p_adj_ = 0.183) between patients with and without hypercholesterinemia.

**Table 6:** Baseline parameter of patients with and without hypercholesterinaemia.

Parameter	Patients with hypercholesterinemia (n = 112)	Patients without hypercholesterinemia (n = 154)	p-value
Age (years)	63 ± 7.1	60 ± 8.5	**0.004**
Diabetes duration (years)	7.1 ± 5.1	5.8 ± 4.5	0.059
Waist circumference (cm)	105 ± 11	106 ± 11	0.492
BMI (kg/m^2^)	30.0 ± 3.9	30.9 ± 4.5	0.103
Sex (m/f)	80/32	94/60	0.079
Heart rate (bpm)	68 ± 10	71 ± 9.2	0.055
systolic OBP (mmHg)	131 ± 13	130 ± 30	0.978
diastolic OBP (mmHg)	78 ± 7.5	79 ± 8.6	0.201
HbA1c (%)	7.1 ± 0.9	7.1 ± 0.8	0.997
HDL-cholesterol (mg/dl)	48 ± 13	46 ± 10	0.315
LDL-cholesterol (mg/dl)	129 ± 34	139 ± 26	**0.009**
Triglycerides (mg/dl)	168 ± 92	170 ± 87	0.854
Non-HDL-cholesterol (mg/dl)	147 ± 41	158 ± 31	**0.013**
hsCRP (mg/l)	1.6 ± 1.7	3.0 ± 3.2	**0.007**
eGFR (CKD-EPI)	90 ± 10	92 ± 12	0.330

Significant values are in bold.

Data are presented as mean ± SD for normal distribution and as median (interquartile range) for not normal distributed data. *OBP* office blood pressure, *hsCRP* high sensitive C-reactive-protein, *eGFR* estimated glomerular filtration rate according to CKD-EPI formula, *UACR* urine albumin creatinine ratio.

## Discussion

In our cross-sectional analysis in patients in the early state of T2DM serum LDL-cholesterol, serum triglycerides, non-HDL-cholesterol, waist circumference and diabetes duration emerged as determinants of arterial stiffness besides the known factors age, sex and systolic office BP. Interestingly, the potpourri of determinants differed depending on the parameter indicating arterial stiffness.

Previous studies identified age and sex as important determinants of arterial stiffness^4^. It is well known that aortic stiffness and arterial pressure are strongly correlated in hypertension with vascular stiffness being both, a cause and a consequence of hypertension^21^. The discussion will therefore focus on the other identified modifiable determinants such as serum LDL-cholesterol, serum triglycerides, WC and diabetes duration.

### Dyslipidemia

Dyslipidemia is a common phenomenon in patients with T2DM^22^. In patients with T2DM the risk of CV disease is greater at any given level of serum cholesterol and its association with hypertriglyceridemia is stronger than in the general population^23^. Dyslipidemia is known to impair endothelium-dependent dilation^22^. Endothelial dysfunction and a reduced contractile response to endothelin-1 trigger vasoconstriction have been previously proposed as important factors contributing to vascular stiffness^24^. Other studies indicate that formation of atherosclerotic plaques, oxidative stress, local and systemic inflammation and low nitric oxide bioavailability might relate PWV and dyslipidemia^25,26^.

In a small prospective cohort study in patients with T2DM increased PWV was associated with dyslipidemia^27^. This is in line with our results. We now also showed that serum LDL-cholesterol and triglyceride levels determine cSBP in patients with T2DM. Interestingly, the diagnosis of hypercholesterinemia did not determine arterial stiffness in our patient population; most likely because the patients with hypercholesterinemia were treated and therefore showed lower LDL-cholesterol compared to the patients without hypercholesterinemia. Patients with T2DM and hypercholesterinemia and/or hypertriglyceridemia even without end-organ damage are known to be at higher CV risk^28^. In patients with T2DM statin therapy has been shown to prevent atherosclerotic cardiovascular disease events and death from coronary heart disease^28^. In addition, statin induced improvement of arterial stiffness has been previously shown for PWV in a small cohort of male patients with T2DM^29^. According to the current guidelines our patient cohort with several risk factors but without overt end-organ damage would qualify for high-intensity statin therapy^28^. Even though our patients were “taking care of their diabetes” as they all participated in RCTs dealing with optimization of their T2DM treatment, only 79 out of the 112 patients (71%) with hypercholesterinemia took statins.

### Waist circumference (WC)

Few data exist on the influence of WC or visceral adiposity on arterial stiffness in patients with T2DM. These data show a relation between WC and PWV in patients with T2DM, but do not investigate a relation between WC and either cSBP or cPP. For example, in a cross-sectional study in patients with T2DM “A Body Shape Index”, which is an indirect measure of visceral adiposity including WC in its calculation, has been shown to be associated with PWV in patients with T2DM^30^ Our results are in line with these findings. However, there are previous data in a large patient population indicating that the effect of increased BP on arterial stiffness is far greater that the influence of visceral adiposity^31^.

### Diabetes duration

In our analysis antihyperglycemic control as judged with HbA1c did not emerge as determinant of arterial stiffness in patients with T2DM. This is in line with results from a large meta-analysis of 102 prospective studies. The meta-analysis showed that fasting blood glucose concentration did not significantly influence vascular disease prediction when added to information about conventional risk factors^4^. However, several preclinical and clinical studies propose the T2DM associated formation of advanced glycation end-products (AGEs) as important factor of vascular stiffening independent of age and other cardio-metabolic risk factors^7^. AGEs form in hyperglycemic environments and decrease arterial wall distensibility. They accumulate in the vessel wall and form cross-links with collagen and elastin fibers^32^. Formation of AGEs typically increases with diabetes duration^4^. To our knowledge this is the first time, diabetes duration has been identified as important determinant of arterial stiffness in our patient cohort.

### Inflammation

Oxidative stress, is an important mechanism mediating increased vascular stiffness in T2DM and known to activate a number of pro-inflammatory pathways^4^. However, measurement of inflammatory markers is not part of the clinical routine work up in patients with T2DM. HsCRP can be considered a common parameter with is widely assessed in clinical routine. Thus, this parameter was chosen as surrogate marker for inflammation in our analysis. We did not see any association between parameters of arterial stiffness and hsCRP in our study cohort. This has to be interpreted with caution as hsCRP only reflects overall inflammation. There are other more specific inflammatory parameters such as IL6, which have not been measured in our analysis.

### Limitations

Our results are based on a fairly small study cohort of patients in the early stage of T2DM. Further investigations in larger study populations are needed. A relevant proportion of patients in our study cohort were treated for the co-morbidities hypertension and hypercholesterinemia. Even though the medication has been kept stable for at least three months before study inclusion, overlapping effects might have influenced our analysis. Finally, the cross-sectional nature of our analysis does not allow to draw conclusions on causal relations.

## Conclusion

In patients in the early state of T2DM arterial stiffness was determined by serum LDL-cholesterol, serum triglycerides, waist circumference and diabetes duration, besides the known factors age, sex and systolic office BP. Treatment of patients in the early stage of T2DM should focus also on these parameters to prevent progression of arterial stiffness and as a consequence cardiovascular mortality.

## Data Availability

The datasets used and/or analysed during the current study are available from the corresponding author on reasonable request.

## Abbreviations

AGE: Advanced glycation end product
BMI: Body mass index
BP: Blood pressure
cDBP: Central diastolic blood pressure
cPP: Central pulse pressure
cSBP: Central systolic blood pressure
CV: Cardiovascular
DD: Diabetes duration
HDL-cholesterol: High density lipoprotein cholesterol
hsCRP: High sensitive c-reactive protein
LDL-cholesterol: Low density lipoprotein cholesterol
PWA: Pulse wave analysis
PWV: Pulse wave velocity
TAG: Triglycerids
T2DM: Type 2 diabetes mellitus
WC: Waist circumference
